# “We should be resourcing their liberation:” a qualitative formative study to guide introduction of a systems engineering intervention at a King County, WA juvenile detention center clinic

**DOI:** 10.1186/s12913-023-09809-6

**Published:** 2023-08-22

**Authors:** Madeline Borges, Lois Schipper, George Gonzalez, Sean Goode, Dorene Hersh, Do-Quyen Pham, Ben Kaplan, Keshet Ronen, Kenneth Sherr, Sarah Gimbel

**Affiliations:** 1 Department of Child, Family, and Population Health Nursing, University of Washington, Seattle, WA USA; 2 King County Department of Adult and Juvenile Detention, Seattle, WA USA; 3 Harborview Abuse and Trauma Center, Seattle, WA USA; 4 CHOOSE 180, Burien, WA USA; 5 Public Health - Seattle & King County, Seattle, WA USA; 6 Department of Pediatrics, University of Washington, Seattle, WA USA; 7 Community Passageways, Seattle, WA USA; 8 Department of Global Health, University of Washington, Seattle, WA USA; 9 Department of Epidemiology, Seattle, WA USA; 10 Department of Industrial and Systems Engineering, Seattle, WA USA

**Keywords:** Youth, Incarceration, Juvenile detention, Care cascades, Systems Analysis and Improvement Approach (SAIA), Systems engineering

## Abstract

**Background:**

There are ongoing efforts to eliminate juvenile detention in King County, WA. An essential element of this work is effectively addressing the health needs of youth who are currently detained to improve their wellbeing and reduce further contact with the criminal legal system. This formative study sought to inform adaptation and piloting of an evidence-based systems engineering strategy – the Systems Analysis and Improvement Approach (SAIA) – in a King County juvenile detention center clinic to improve quality and continuity of healthcare services. Our aims were to describe the priority health needs of young people who are involved in Washington’s criminal legal system and the current system of healthcare for young people who are detained.

**Methods:**

We conducted nine individual interviews with providers serving youth. We also obtained de-identified quantitative summary reports of quality improvement discussions held between clinic staff and 13 young people who were detained at the time of data collection. Interview transcripts were analyzed using deductive and inductive coding and quantitative data were used to triangulate emergent themes.

**Results:**

Providers identified three priority healthcare cascades for detention-based health services—mental health, substance use, and primary healthcare—and reported that care for these concerns is often introduced for the first time in detention. Interviewees classified incarceration itself as a health hazard, highlighting the paradox of resourcing healthcare quality improvement interventions in an inherently harmful setting. Fractured communication and collaboration across detention- and community-based entities drives systems-level inefficiencies, obstructs access to health and social services for marginalized youth, and fragments the continuum of care for young people establishing care plans while detained in King County. 31% of youth self-reported receiving episodic healthcare prior to detention, 15% reported never having medical care prior to entering detention, and 46% had concerns about finding healthcare services upon release to the community.

**Conclusions:**

Systems engineering interventions such as the SAIA may be appropriate and feasible approaches to build systems thinking across and between services, remedy systemic challenges, and ensure necessary information sharing for care continuity. However, more information is needed directly from youth to draw conclusions about effective pathways for healthcare quality improvement.

**Supplementary Information:**

The online version contains supplementary material available at 10.1186/s12913-023-09809-6.

## Background

The practices of incarcerating and detaining children and adolescents cause well-documented harm to individual and community well-being [1–4]. Put simply, youth have a better chance to thrive if they are not in contact with the legal system. Furthermore, Black, Indigenous, and young People of Color are overrepresented in detention facilities, including in Washington State [5], due to generations of racist policies and practices that permeate educational, legal, and social systems. A rich body of evidence details these systemic biases, including prejudiced decision-making by individual actors in the legal system [6, 7], disproportionate levels of criminalization and surveillance in communities of color compared to white communities [8, 9], and punitive disciplinary procedures in schools that create a “school-to-prison pipeline” predominantly targeting young people of color [10].

In Washington State, there are ongoing efforts to promote a public health approach [11] to eliminate juvenile detention. King County’s strategic plan includes a ‘Road Map to Zero Youth Detention’ to guide investments and policies that support families and reduce the number of youth involved in the legal system [12]. An essential element of this roadmap is to ensure quality care for the health needs of young people who are currently detained [13] in order to improve their well-being and prevent future contact with the legal system [14–17].

Robust systems engineering methods [18] can optimize care cascades, which are linked, sequential steps in a care pathway of health services [18]. These methods allow us to 1) identify the main drivers for system inefficiency, 2) support provider decision-making to prioritize interventions, and 3) improve integration of services to meet the diverse clinical needs of patients [18]. Low-cost, systems-level interventions are effective and efficient approaches to improve linked cascade services. These interventions may be effective for improving the clinical care of detained youth, addressing individual- and system-level barriers, improving flow through multiple care cascades, and ultimately improving patient-level outcomes. The *Systems Analysis and Improvement Approach* (SAIA) [19] is an evidence-based implementation strategy, designed by and for frontline health workers, that combines systems engineering tools into a multi-step, facility-level package to improve understanding of gaps, guide identification and prioritization of low-cost workflow modifications, and iteratively test and redesign these modifications [20, 21].

This formative study sought to inform adaptation and piloting of the SAIA in a King County, WA juvenile detention setting to improve the quality and continuity of healthcare services by describing 1) the priority health needs of young people who are involved in Washington’s criminal legal system, and 2) the current system of healthcare for young people who are detained.

## Methods

We used qualitative methods to inform a systems view of the primary healthcare cascades for youth who are detained in King County, conducting individual interviews with health and social service providers serving young people involved in the criminal legal system. Additionally, we obtained de-identified internal summary reports of quality improvement discussions held during routine health education classes by clinic staff with young people who were detained at the time of data collection.

### Setting

The King County juvenile detention center had a total of 416 bookings and 387 releases in 2022, with an average daily population in secure detention of 34.2 [22]. The average length of stay for youth in detention under juvenile court jurisdiction was 23.2 days and for youth under adult court jurisdiction was 282.6 days [22]. All youth detained at the King County juvenile detention center are under the age of 18—youth that turn 18 in custody are transferred to adult detention on their 18^th^ birthday. The most recent demographic report of youth in King County detention showed that in 2020, 28% of youth admitted to detention were female and 72% were male [23]. The same report showed that 25.7% of detained youth were White, 21% were Latinx, 42.1% were Black, 2.1% were Native American/Alaska Native, and 8.6% were Asian American/Pacific Islander [23]. In 2020, 65.9% of youth in secure detention were admitted for alleged or adjudicated felonies, 26.1% for alleged or adjudicated misdemeanors, 4.6% for a violation of a court order related to a “criminal matter” (e.g. probation violations), and 1.7% were admitted for a violation of a court order related to a non-offender reason (e.g. dependency cases, truancy petitions, and others) [23]. The authors note here that policies related to COVID-19 contributed to drastic reductions in admissions to secure juvenile detention and changes within the juvenile legal system during 2020.

### Interview participant selection

We used a purposive sampling approach, recruiting participants with experience in providing 1) healthcare services in a juvenile detention setting, or 2) community, social, or legal services for young people who are involved in the criminal legal system. Participants were included if they were 18 years of age or older and had worked at a health or social service organization which supports health care, treatment, or prevention for previously or currently incarcerated youth. Participants were excluded if they were under the age of 18, did not speak English, or did not consent to participate in the interview.

We conducted stakeholder mapping with a key informant at the juvenile detention center to determine stakeholders with knowledge of young people’s health concerns before, during, and after detention. Based on our stakeholder mapping exercise, we identified potential interview participants and contacted them via email. Willing participants were consented via Zoom and email.

### Interview data collection

The last author (SG) and first author (MB) conducted the interviews. Interviews were conducted in English over Zoom between May 2021 and June 2021. At the beginning of the interview, we emphasized that participants could skip or refuse to answer any question without penalty or need for clarification. Prior to starting interview questions, we reminded participants to let us know if they were uncomfortable at any time. Additionally, we stayed alert to signs of discomfort in participants and paused the interview if participants seemed uncomfortable. We conducted nine 45-minute in-depth interviews with stakeholders experienced in providing court-mandated mental health assessment services, probation supervision, child welfare services, community-centered alternatives to the criminal legal system, healthcare in community shelters for people living unhoused, and providing healthcare in a Washington State juvenile detention setting. Respondents were not asked about any demographic information. We used a semi-structured interview guide to understand participants’ perspectives on the primary health concerns of young people who are detained in King County. All interviews were recorded.

### Interview data analysis

Interviews were transcribed verbatim by MB and all identifying information was removed. SG and MB coded transcripts using Microsoft Word. We first read transcripts for comprehension. We developed a coding frame deductively based on the research questions of interest (See Additional File 1). After the initial review of all transcripts, making notes of recurrent ideas and responses, we added four additional codes using an inductive approach: *Violence health concern* was added after violence was referenced three times as a disease or health concern*; Polypharmacy/Overdiagnosis* was added after the overdiagnosis and overmedication of youth was brought up by two participants; *Communication* was added after five participants referenced communication barriers in the continuum of care for youth people who are detained; and *Political environment* was added after three participants referenced the politicized nature of juvenile detention in King County, WA.

### Secondary quantitative data sources

During the study period, health care staff at the juvenile detention clinic conducted several group discussions with 13 youth as part of an internal clinic quality improvement procedure. Discussions occurred during a routine health education module and were designed to assess healthcare access prior to detention, as well as care youth received at the detention center’s clinic. Youth engaged in discussion with healthcare workers about past experiences with healthcare, current experience at the detention clinic, and concerns about healthcare upon release. Clinic staff took notes during discussions to assist with quality improvement efforts, including de-identified summary statistics of the number of youth who mentioned access to healthcare prior to detention, the number of youth who reported utilizing care while detained, and the number of youth who reported healthcare concerns upon their anticipated release from detention. We used these secondary summary data to triangulate emergent themes from interviews.

## Results

### Quantitative results

A summary of quantitative data is presented in Table 1. Demographic information about discussion group participants was not routinely included in summary statistics. Therefore, we were unable to collect demographic information about participants. Among the 13 youth who participated in discussions with clinic staff during health education classes, 54% (7) self-reported regular access to healthcare, 31% (4) self-reported episodic care through emergency rooms or school-based health clinics, and 15% (2) self-reported never having medical care prior to entering detention. 100% (13) self-reported accessing healthcare during their detainment. 46% (6) of young people self-reported concerns about finding healthcare services upon release to the community from detention.

**Table 1:** De-identified summary from discussions between health clinic providers and youth who are currently detained

*Healthcare access*	*% (n) of youth self-reporting*
Regular^a^ access to healthcare prior to entering detention	54% (7)
Episodic access to healthcare through emergency rooms and/or school-based health clinics prior to entering detention	31% (4)
Never accessed healthcare prior to entering detention	15% (2)
Have accessed healthcare while detained	100% (13)
Have concerns about accessing care after leaving detention	46% (6)
Have no concerns about accessing care after leaving detention	54% (7)

^a^defined by youth as annually or “as needed”

### Qualitative results

Three key themes emerged from qualitative interviews related to the priority health needs of young people who are involved in Washington State’s criminal legal system, and the current system of healthcare for young people who are detained: 1) Providers define three distinct priority healthcare cascades for youth whose behavior has been criminalized in King County; 2) Carceral systems cause and exacerbate health concerns; 3) Siloing of care and communication across community- and detention center- based services impede linkages to care upon release.

#### Providers identify three distinct priority healthcare cascades for youth whose behavior has been criminalized

Mental health, substance use, and primary health care emerged as the priority care cascades for youth whose behavior has been criminalized in Washington State. Participants described supporting young people with high levels of trauma, Adverse Childhood Experiences (ACEs) [24]), and violence in their lives, contributing to high rates of mental health concerns and substance use.

*I think number one is trauma and other ACEs […] and I would put substance use as a very, very close second […] they are so intimately linked. […] And incredibly high rates of chronic disease. And it’s kind of all types of things I would have expected. […] But we also see unusual neurological disorders, autoimmune disorders. […] I've come to believe that […] chronic stress contributes to many, many types of medical and health issues in youth. -*1009

While mental health concerns were mentioned in all interviews as a primary concern for young people involved in the criminal legal system, a thematic nuance emerged regarding a pattern of overdiagnosis and polypharmacy related to behavioral health conditions among youth. Participants acknowledged observing a pattern of clinicians pathologizing and medicating youthful behaviors related to trauma:

*“Mental health - this is where I think there's almost an over-diagnosis of kids [...] every child gets a label [...] and then they have a backpack full of diagnoses that don't really help you understand what's going on with the child and often point people in the wrong direction. [...] And kids who are basically acting out in their environment are often, you know, getting labeled ‘conduct disorders.’ [...] If you looked at their circumstances...It makes sense, what they're doing.”*- 1003

Additionally, participants described seeing little healthcare access and a need for primary healthcare among youth involved in Washington’s criminal legal system. Participants noted that a main driver for lack of healthcare access is limited material resources straining families’ ability to prioritize healthcare.

*“In terms of our general things, many of our youth come from families with limited resources. And so, in addition to all the trauma that they're experiencing, they don't really have consistent access to primary care or preventative health. They're more likely to engage in sexual activity, yet less likely to have consistent contraception. [...] It’s during their stay with us [at the juvenile detention center] that we're catching them up on things like immunizations, or dental screening vision screening”* -1007

#### Carceral systems cause and exacerbate health concerns

Another theme we identified is the classification of carceral systems as a health hazard, causing harm to an individual’s health by exacerbating existing health concerns and directly causing additional trauma. Participants highlighted the importance of context when providing healthcare in a detention facility and the paradox of resourcing healthcare or harm reduction in an environment that is inherently harmful to a young person’s health:

*“The process of incarceration exacerbates any health concerns that a young person may have prior to entering. […] the process of criminalizing behavior, the process of detaining a young person whose behavior has been criminalized, the process of intake, the process of putting them behind bars.*

*I think all of that isn't trauma-informed and none of that actually helps heal and it only does more to cause harm. So, just from a public health practice…It seems regressive to want to focus on the health of a young person, while simultaneously putting them through a process that causes harm.”* -1004

#### Siloing of care and communication across community- and detention center-based services

The third theme we identified is a pattern of siloed care and communication across community- and detention center-based health services that impedes linkages to care upon release. While entities serving health-related needs strive to optimize their services, siloing within and between care sites results in redundancies and inefficiencies. Though appropriate services may exist within individual organizations serving youth, fractured communication and collaboration across entities drives the majority of inefficiency and poor system outcomes.

*“I think the communication, the ability to communicate more openly and more thoroughly about individual health needs [...] could be better if folks agreed it was a priority. ”* -1006

*“[The provider] that I was working with on one young person…she had a whole plan, and at first I was like “whoa what is going on because I'm working on this whole other thing over here, it's going to get him to treatment so can we like…get united around messaging.” Because it was around Suboxone and so then the kid interpreted that that he didn't need to go to treatment [...] there just isn't a lot of cross communication.”* -1008

Participants highlighted concern around a broken continuum of care for young people that establish mental and physical health care plans while detained. As our quantitative data illustrates, for many youth involved in the criminal legal system, healthcare is introduced for the first time in the juvenile detention context. Upon release to community services, the burden of navigating care falls on youth and their families:

*“The same enthusiasm we bring in incarcerating young people, we should be resourcing their liberation in the same manner. […] What exists [today] is that you have somebody in probation that says okay well here's somebody you can connect with. And if you don't connect with them, then you're likely to get locked up again, or if they're no longer on probation, they are released to their family with some recommendations that people they can connect with. And there's not the same level of investment in a young person's possibility as much as we're invested in a young person as a problem.” -*1004

## Discussion

This study sought to inform an adaptation of the SAIA in a King County, WA juvenile detention setting by describing 1) the priority health needs of young people who are involved in the criminal legal system, and 2) the current system of care for young people who are detained. Providers serving young people defined three priority care cascades for youth who are involved in the criminal legal system: mental health, substance use, and primary health care. We also found that multiple agencies, organizations, and institutions interact with youth in King County beyond the walls of the detention center. While these entities strive to optimize their services, siloing within and between care sites results in redundancies and inefficiencies. According to providers, fractured communication and collaboration across entities drives the majority of inefficiency and poor outcomes for youth, specifically impacting a broken continuum of care for young people that establish mental and physical health care plans while detained. Qualitative findings converged with quantitative data in our study, suggesting that healthcare is often introduced for the first time in a setting of confinement and trauma for young people who are detained. Upon release to community services, healthcare navigation is a burden that falls on youth and their families who often have little trust in providers and significant challenges that impede their ability to prioritize healthcare navigation.

Many of our findings reflect those described previously in the literature. Previous studies have found that youth incarcerated in the United States had variable levels of healthcare access prior to their confinement [25] and higher rates of physical and mental health concerns, including substance use disorder, than young people who are not incarcerated [25–27]. Authors have attributed this pattern to the fact that people who are involved in the criminal legal system often live in under-resourced, highly-policed neighborhoods or communities, contributing to disparities across social determinants of health [26]. This is compounded by lack of access to healthcare, resulting in higher levels of health concerns. Previous studies have also found that incarceration contributes to increased or exacerbated health concerns after detention [3, 28].

While many of our findings converge with the existing literature, little research has focused on health and continuity of medical care upon release. In a 2020 systematic review of the scholarly literature, Barnert and colleagues found only 10 research articles on health status or care access for youth reentering communities from confinement [13]. The dearth of research in this area aligns with one of our participants’ observations that, systemically, “there’s not the same level of investment in a young person's possibility as much as we're invested in a young person as a problem.”

There are notable limitations to this study. First, our study does not include perspectives directly from young people who are currently or were previously detained. Due to systemic challenges obtaining informed consent from caregivers and legal guardians to interview youth directly, we relied on qualitative information from adult providers serving youth. This limits our ability to confirm their perceptions with the perspectives of young people living the experience of healthcare in a carceral institution, and mirrors a pattern [29] of leaving youth out of decision-making conversations around their care. Additional data are needed from youth to make firm conclusions about areas for systems improvement. Secondly, our secondary quantitative data relied on youth self-reporting their experiences with healthcare to clinic staff and providers’ synthesis of youth self-reports. This data collection method carries opportunity for human error, bias, and resulting inaccuracy. Additionally, our data comes from one jurisdiction, preventing us from generalizing our findings beyond this study setting. However, our findings mirror those of previous studies, suggesting that these results are likely relevant elsewhere.

Despite these limitations, there were several strengths to the study. First, this is a novel setting for adapting the SAIA strategy and offers insight into both the role of systems engineering in carceral settings and the rarely researched complex systems youth face when they are released from detention. Second, we conducted in-depth interviews with several actors with diverse roles and extensive experience working with youth engaged in the criminal legal system. This allowed us to collect nuanced information from multiple angles both inside and outside the criminal legal system. Third, using a semi-structured interview format allowed the flexibility to discover emergent themes beyond our initial research goals.

Our results have several implications for implementing the SAIA and other systems engineering interventions in the juvenile detention setting. Our findings highlight the irony that for many young people, care processes for health concerns are introduced or continued within a setting that is ultimately harmful to their health. The SAIA’s unique package of systems engineering tools may be appropriate to iteratively improve the delivery of care in complex systems [30] of juvenile detention-based healthcare and improve outcomes for young people who are released from confinement through two mechanisms: 1) Reducing further harm that occurs while youth are detained by centering patient experiences of health care in detention-based clinics and effectively addressing their healthcare needs; 2) Resourcing and optimizing release planning support systems by addressing siloed care and communication pathways to improve linkages and accountability across services. Cascade analysis presents an opportunity for detention-based health workers and managers to utilize their routine data, visualize the impact of their work, and identify systemic bottlenecks. Complemented by process mapping and continuous quality improvement, this approach allows frontline workers to holistically understand causes of health delivery system failure and identify and prioritize solutions [31], creating transformative potential within complex systems such as carceral settings.

## Conclusions

We found that providers serving youth engaged in the criminal legal system define mental health, substance use, and primary health care as the priority care cascades for young people who are involved in Washington’s criminal legal system. We also found that young people self-reported to clinic staff little to no experience receiving regular healthcare services prior to entering detention, receiving healthcare services while detained, and concerns about finding healthcare services upon release to the community from detention. Providers describe a similar pattern of limited prior care access among youth and view carceral systems as hazardous to health, causing harm to individuals’ health by exacerbating existing health concerns and directly causing additional trauma. Providers also describe siloed services across detention- and community-based settings, impeding care continuity for youth who establish mental and physical care plans while detained. Strategies that work to align systems of care, reduce the burden of care navigation on youth and families, and increase efficiency across care cascades are necessary in this context. The SAIA is a potentially appropriate and feasible approach to build systems thinking across and between services, remedy systemic challenges, and ensure necessary information sharing for care continuity. However, more nuanced information is needed from youth themselves in order to draw conclusions about effective pathways for systems change. Future directions for this work include research methods that deeply engage youth with lived experience of the criminal legal system to determine appropriate methods for improving their care.

### Supplementary Information

**Additional file 1.  **Coding frame used for qualitative data analysis.

## Data Availability

The datasets generated and/or analyzed during the current study are not publicly available in order to protect participants’ anonymity but are available from the corresponding author on reasonable request.

## Abbreviations

ACE: Adverse Childhood Experiences
SAIA: Systems Analysis and Improvement Approach
